# Psychological constraint on unethical behavior in team-based competition

**DOI:** 10.3389/fpsyg.2023.1274414

**Published:** 2023-11-14

**Authors:** Yi Zhu, Lijing Zheng, Yu Hu

**Affiliations:** ^1^School of Early-Childhood Education, Nanjing Xiaozhuang University, Nanjing, Jiangsu, China; ^2^Business School, Sun Yat-sen University, Shenzhen, Guangdong, China; ^3^Research Institute of Social Development, Southwestern University of Finance and Economics, Chengdu, Sichuan, China

**Keywords:** intergroup competition, lying behavior, collective efficacy beliefs, immoral behavior, group identification

## Abstract

A growing body of research contributes to our knowledge about unethical behavior. However, very little is known about how group-based competition shape members’ unethical behavior. Building on social learning theory, we conducted three studies to reveal how group-based competition may affect individual’s unethical behavior for their team. Study 1 and 2 are laboratory experiments in which participants were randomly assigned into groups of three members and engaged in group-based competition (or engaged in individual-based competition in an individual context) with monetary incentives. Different from individual-based competition where mean number of unethical behaviors for the self in the losing condition was larger than that in the winning condition, in group-based competition mean number of unethical behaviors in favor of group between the winning and the losing condition was not significantly different. Both studies also showed that there are less unethical behaviors in the group-based competition than in the individual-based competition. Study 2 further revealed that collective efficacy negatively associated with mean number of unethical behaviors in group-based competition. Study 3 was a field study with employees from bank subsidiaries working as teams, and results from their self-reported data confirm the relationship between collective efficacy and unethical behaviors observed in Study 2. Together, these results suggest that collective efficacy has the effect of curbing unethical behavior in group-based competition, thus contributing to the understanding of group-based experience on unethical behaviors.

## Introduction

Groups are widely utilized to organize people toward collective goals (Kozlowski and Ilgen, 2006). In the course of goal pursuit, there are usually multiple groups striving for incompatible goals (e.g., companies compete for market share, football teams compete for championship). One’s progress in attaining desired outcomes decreases others’ probability of goal attainment, so group members in this context will be motivated to gain or maintain advantages (Deutsch, 1949; Pruitt, 1998; De Dreu, 2010; Garcia et al., 2013).

Competition not only can promote competitiveness, leading to improved group performance (Pike et al., 2018), but also can provoke unethical behavior (Goette et al., 2012; Bennett et al., 2013; Kilduff et al., 2016). Although group-based competition are pervasive in human society, empirical research examining unethical behavior in this context is still limited. The present research hereby explores whether unethical behavior in group-based competition differs from that in individual-based competition, and tries to reveal how social bonding in groups affects individuals’ unethical behavior.

## Competition

Competition is interaction processes in which individuals vie for goals or resources, with one party’s goal pursuit impeding another party’s goal striving (Deutsch, 1949).

Engaging in competition may entail increased physiological arousal (Neave and Wolfson, 2003; To et al., 2018), result in risk-taking behaviors (To et al., 2018) and heightened motivation to outperform their opponents (Yip et al., 2018).

When individuals are outperformed by opponents, such experience may hurt their identity (Aquino and Douglas, 2003; Leavitt and Sluss, 2015) and give rise to discouragement and lowered self-satisfaction (Bandura, 1991). According to social learning perspective, individuals in this situation would apply their moral standards more leniently (Bandura, 1999; Sharma et al., 2014) and tend to morally disengage from self-regulation (Bandura, 1991, 1999). Thus the situation of being disadvantageous would inflame unethical impulse, triggering unethical behaviors aiming at gaining advantage (Pierce et al., 2013). Based on the above reasoning, we could expect an enhanced value competition losers would place on retaining advantages, leading to an increase in unethical behavior, as compared to winners.

## Group-based competition and unethical behavior

Individuals have the innate need to affiliate with social groups (Baumeister and Leary, 1995), and to identify with their groups (Tajfel, 1982; Ashforth and Mael, 1989). In group-based competition contexts, because individuals are concerned about their relative group outcomes, they are apt to participate in unethical behaviors toward competitors to maintain group advantage (Halevy et al., 2010; Goette et al., 2012). Unethical behaviors targeting at improving group interests rather than the self are coined *pro-group unethical behaviors* (Thau et al., 2015) and *unethical pro-organizational behavior* (Umphress and Bingham, 2011). Such unethical behaviors have been consistently observed in recent studies (Molinsky and Margolis, 2005; Gino and Pierce, 2009; Umphress et al., 2010; Umphress and Bingham, 2011; Shalvi and De Dreu, 2014; Thau et al., 2015; Chen et al., 2016; Fehr et al., 2019; Jiang et al., 2022). It is suggested that individuals conduct pro-group unethical behaviors with the purpose of being included by their groups (Thau et al., 2015) or maintaining exchange relationship with their groups (Umphress et al., 2010; Umphress and Bingham, 2011).

In many work groups, task jointness and coordination produce a sense of shared responsibility which fosters affective ties to groups (Thye et al., 2019), promote ingroup cohesiveness (Deutsch, 1949; Stein, 1976; Tajfel and Turner, 1979) and cooperation (Erev et al., 1993; Bornstein et al., 2002). Individual members embedded in such cohesive relational network act as valuable resources to each other, as their interactions consist of such features as mutual aid, reciprocal norms, and interpersonal trust that enable individuals to act together effectively to pursue collective goals (Putnam, 1993). According to Bandura’s social cognitive theory (Bandura, 2000), the interdependence of individual member functioning and members’ shared beliefs in their collective power to produce effects provide a basis for the development of collective efficacy belief—belief in the collective power to produce desired outcomes (Gibson, 1999; Bandura, 2000; Kozlowski and Ilgen, 2006). This belief has positive impact on psychological wellbeing. For instance, it associates with resilience to impediments and setbacks (Bandura, 2000), serves as buffer of stressor-strain relations (Jex and Bliese, 1999; Tucker et al., 2013; Esnard and Roques, 2014), and reduces anxiety (Salanova et al., 2003). Thus collective efficacy belief developed in group-based competition is helpful in buffering stress produced in individuals’ interaction with rivals, leading to a more benign appraisal of stressful events in competitive interaction (e.g., group-based disadvantage), as suggested by previous literature (Kawachi and Berkman, 2001).

On the other hand, individuals like to think of themselves as moral, so conducts that are in line with their moral standards build their sense of self-worth, whereas behaving in ways that violate their moral standards are detrimental to their self-worth and may cause self-condemnation (Bandura, 1999). Since individuals who engage in joint tasks can count on other group members’ expertise and efforts, collective efficacy belief would have the effect of mitigating individuals’ impulse to behave in ways that will violate their moral standards in group-based competitions. Indeed, recent work suggests that individuals sometimes are aversive to unethical behavior when it advances their own interests because they want to maintain their positive view of self-concept (Mazar et al., 2008; Mead et al., 2009; Shalvi et al., 2011a,b).

Therefore, group-based competitions would have differential impact on behaviors, as compared to individual-based competitions where outcomes are solely determined by personal performance. We could expect a mitigated motivation to behave unethically in group-based competitions, compared with that in individual-based competitions, and a negative association between collective efficacy belief and unethical behavior in group-based competitions.

*Hypothesis 1:* There are more unethical behaviors in the losing condition than in the winning condition in individual-based competitions.

*Hypothesis 2:* There are less unethical behaviors in the group-based competitions than in the individual-based competition.

*Hypothesis 3:* There is a negative association between collective efficacy belief and unethical behavior in group-based competitions.

## Overview of the present research

To test the hypotheses, the present study focused on unethical behavior after winning or losing a competition. Study 1 and 2 are laboratory experiments in which participants were assigned into groups of three members and engaged in group-based competitions (or individual-based competitions in an individual context) with monetary incentives. Participants were instructed to engage in two consecutive competitions with the same competitor, namely repeated team-based competition (RTB) or repeated individual-based competition (RIB). In Study 1, winning or losing outcome was determined by participants’ true performances. The authors compared unethical behaviors between the winning and the losing conditions and between the RTB and the RIB. In Study 2, participants were randomly assigned to a winning or a losing condition. The purposes of Study 2 were to replicate findings in Study 1, and test the relationship between collective efficacy and pro-group unethical behavior. Study 3 was a survey study with employees from bank subsidiaries working as teams, to garner empirical evidence for ecological validity of our conclusions.

## Study 1

### Methods

#### Participants

A total of 132 native Chinese-speaking undergraduate students from a university in southwest China (44% male; *M*_*age*_ = 20.77, *SD*_*age*_ = 1.58; *N*_*RTB*_ = 72, *N*_*RIB*_ = 60) participated in this experiment. The experiment employed a 2 (competition mode: individual-based competition vs. team-based competition) × 2 (competition outcome: win vs. loss) between-subjects design. Gender ratio was balanced in each cell. In the absence of a suitable estimate of expected effect size, the sample size was derived from a previous study using a similar protocol (Schurr and Ritov, 2016). Three participants in RIB were dropped out from data analysis because of equal performance in a dyad in the first competition (*N* = 2) or skepticism about the competition outcome (*N* = 1).

#### Experimental procedure

The experiment was conducted in a computer lab. Upon arrival, participants read and signed the informed consent, and were ushered to sit in front of their computer screens. Before the onset of the experiment, an experimenter introduced all the experimental tasks, and then participants proceeded to complete each task under the guidance of the experimenter.

**FIGURE 1:**
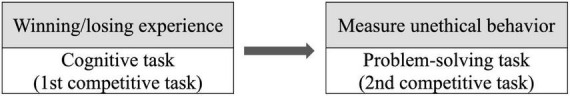
Experimental procedure.

Participants were instructed to engage in two consecutive competitions with the same competitor for bonus (see Figure 1). In RTB, participants were informed that they had been randomly assigned into teams of three and each team had been randomly paired with another team to engage in two consecutive competitions. In RIB, participants were informed that they had been randomly paired with another individual to engage in two consecutive competitions. They first engaged in a cognitive task (the 1st competition) in which they experienced winning or losing, and then competed with the same competitor in a problem-solving task (the 2nd competition which was also used to track their unethical behavior). They participated in exchange for 20 RMB (2.74 USD) plus 15 RMB (2.05 USD) bonus for winning each competition. To minimize social influences, participants were anonymous to one another. Finally, participants indicated whether they were skeptical about the outcomes they received in the two competitive tasks.

#### Cognitive task

**FIGURE 2:**
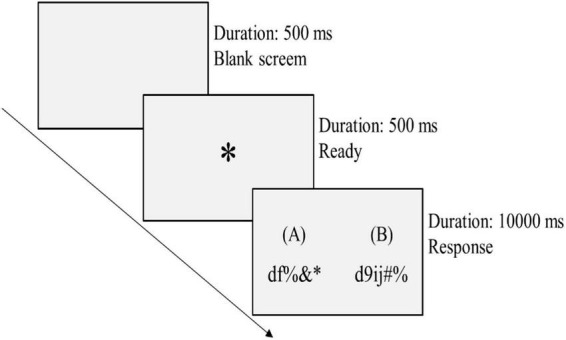
The cognitive task. *Appears in the middle of the screen for 500 ms between the blank screen and the response screen to calibrate visual fixation on the screen.

This was the first competition task used to elicit winning or losing experience. There were 10 practice trials and 50 formal trials (see Figure 2). In each trial, participants identified the number of identical characters between two random character strings shown on their screen (e.g., “df%&*” and “d9ij#%”) and responded by pressing a number key within 10 s (press “2” for the above sample). The number of characters in each string ranged from 4 to 11, and the number of identical characters between each pair of strings ranged from 0 to 9. There was no feedback after each trial; participants were shown on their screen their total correct responses once completing all the trials.

The cognitive task was completed in private and after all participants completed this task, two experimenters kept record of their scores. Participants were informed that winning and losing outcomes were determined by their actual performance and those whose total number of correct responses exceeded their competitor were winners. Information regarding their own score, team total score (only in RTB), competitor’s total score, competition outcome (i.e., win/loss), and reward (i.e., 0/15RMB) was filled on the first page of questionnaires distributed to them according to their seat numbers.

#### Problem-solving task

To track unethical behavior, the authors created a Chinese version of anagram task. Inspired from standard anagram task in which participants create words from different series of seven letters under time pressure (Gino and Margolis, 2011; Thau et al., 2015; Welsh et al., 2015), participants in the present study were asked to find as many four-character idioms as possible based on 200 Chinese characters spread in a 10 × 20 table within 60 s. They then wrote on an answer sheet the number of idioms they found, giving them the opportunity to over-report their performance. Unbeknownst to them, the problem was unsolvable except for two obvious idioms intentionally aligned in the center of the table (i.e., “根深蒂固,” “坐井观天”). All participants should be familiar with the two idioms. Participants could win 15RMB bonus if the number of idioms they reported exceeded that of their rival, so they had the motivation to over-report. Following Thau et al. (2015), the number of idioms over-reported constituted our measure of unethical behavior.

### Results

**FIGURE 3:**
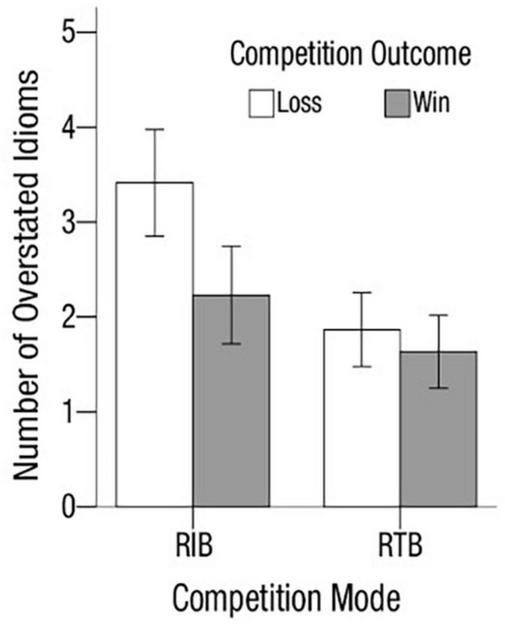
Study 1: mean number of idioms overstated by condition. Error bars represent standard errors.

Figure 3 displays the mean number of idioms over-reported by condition. The analysis yielded a significant main effect of competition outcome [*M*_*loss*_ = 1.95, *SD*_*loss*_ = 3.54 vs. *M*_*win*_ = 0.97, *SD*_*win*_ = 1.61; *F*_(1, 125)_ = 5.09, *p* < 0.05, η_*p*_^2^ = 0.04]. Further analyses showed that the number of idioms over-reported in the losing group was significantly larger than that in the winning condition in RIB [*M*_*loss*_ = 3.14, *SD*_*loss*_ = 4.69 vs. *M*_*win*_ = 1.29, *SD*_*win*_ = 1.96; *F*_(1, 55)_ = 3.74, *p* = 0.058, η_*p*_^2^ = 0.06], thus supporting Hypothesis 1. This difference was not statistically significant in RTB [*M*_*loss*_ = 1.00, *SD*_*loss*_ = 1.79 vs. *M*_*win*_ = 0.72, *SD*_*win*_ = 1.26; *F*_(1, 70)_ = 0.581, *p* = 0.448]. Results also provide support for Hypothesis 2 by showing a main effect of competition mode—the number of idioms over-reported in RIB was larger than that in RTB [*M*_*RIB*_ = 2.23, *SD*_*RIB*_ = 3.70 vs. *M*_*RTB*_ = 0.86, *SD*_*RTB*_ = 1.54; *F*_(1, 125)_ = 8.18, *p* < 0.01, η_*p*_^2^ = 0.06]. There was no interaction effect.

Findings in Study 1 suggest that group or team has an effect of mitigating the demonstration of unethical behavior. We reasoned that unethical behavior in team-based competitions could be constrained by collective efficacy. However, Study 1 did not provide direct evidence for this point, thus Study 2 was designed to replicate findings in Study 1 and to shed light on the relationship between collective efficacy and unethical behavior in team-based competition.

## Study 2

### Methods

#### Participants

A total of 120 students (45% male; *M*_*age*_ = 21.23, *SD*_*age*_ = 1.76; *N*_*RIB*_ = 60, *N*_*RTB*_ = 60) participated in this study. Experimental protocol was similar to the one used in Experiment 1. Nine participants in RIB were dropped out from data analysis because of equal performance in a pair of students in the cognitive task (*N* = 2), or skepticism about the competition results (*N* = 2), or computer glitch (*N* = 5).

#### Experimental procedure

Experimental procedure was identical to Study 1 with the following exceptions: (1) Winning and losing in the cognitive task were determined by chance, allowing to control individual difference variables (e.g., cognitive ability) that could affect their unethical behaviors. (2) After the practice of the cognitive task, participants in RTB completed items measuring their collective efficacy and group identification. According to the minimal group paradigm (Tajfel, 1970), merely being categorized into an experimental group is sufficient to induce favoritism to the ingroup and discrimination against an out-group. These measures can reveal how collective efficacy and group identification link to unethical behavior after winning or losing a competition. In addition, the authors also measured affectivity as a control variable.

#### Measure

In RTB, competition outcomes are determined by collective efforts, and each member has no direct control about team outcome they desire. Under such circumstance, individuals turn to collective agency—relying on peers’ expertise and efforts in group tasks (Bandura, 2000). Therefore, collective efficacy may play an important role in shaping their behaviors. To reveal the relationship between collective efficacy and unethical behavior, the authors included a four-item scale adopted from Jex and Bliese (1999), α = 0.85 (e.g., “I have real confidence in my team’s ability to perform its mission”). Responses were given on a 9-point scale ranging from 1 (*strongly disagree*) to 9 (*strongly agree*). Previous literature suggests that group identification associates with unethical behavior in favor of in-groups (Umphress et al., 2010; Umphress and Bingham, 2011), so the present study assessed participants’ team identification with a four-item scale modeled from Doosje et al. (1995), α = 0.86 (e.g., “I am glad to be a member of our team”). Responses were given on a nine-point scale ranging from 1 (*not at all*) to 9 (*very much*). An affectivity scale (Watson et al., 1988) was used to measure participants’ feelings after the first competition (Cronbach’s α:0.84 for Positive Affect subscale, 0.88 for Negative Affect subscale). Participants indicated to what extent they had experienced the feelings depicted at the present moment (e.g., “hostile,” “excited”) on a five-point scale ranging from 1 (*very slightly or not at all*) to 5 (*extremely*).

### Results

**FIGURE 4:**
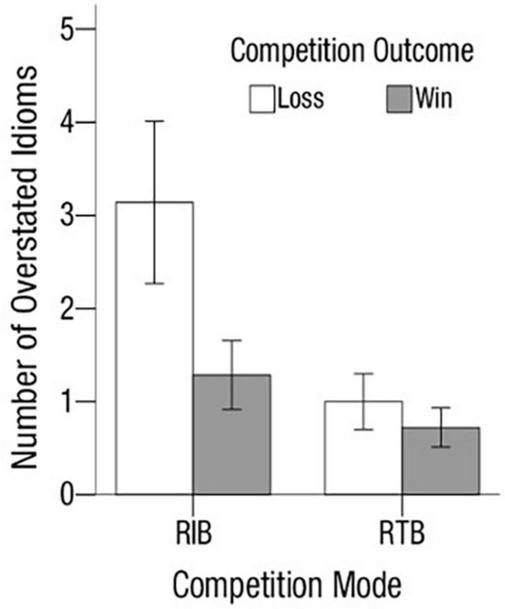
Study 2: mean number of idioms over-reported by condition. Error bars represent standard errors.

Figure 4 displays the mean number of four-character idioms over-reported by condition. Results showed a main effect of competition mode [*M*_RIB_ = 3.35, *SD*_RIB_ = 4.61 vs. *M*_RTB_ = 1.75, *SD*_RTB_ = 2.10; *F*_(1, 107)_ = 6.28, *p* < 0.02, η_p_^2^ = 0.06], and a main effect of competition outcome [*M*_loss_ = 3.11, *SD*_loss_ = 3.93 vs. *M*_win_ = 1.88, *SD*_win_ = 3.06; *F*_(1, 107)_ = 4.26, *p* < 0.05, η_p_^2^ = 0.04]. There was no interaction effect. Furthermore, the number of idioms over-reported in losing condition was larger than that in the winning condition, and the difference was significant in RIB [*M*_loss_ = 4.60, *SD*_loss_ = 5.00 vs. *M*_win_ = 2.15, *SD*_win_ = 3.93; *F*_(1, 51)_ = 3.79, *p* = 0.057, η_p_^2^ = 0.07], but not in RTB [*M*_loss_ = 1.87, *SD*_loss_ = 2.13 vs. *M*_loss_ = 1.63, *SD*_loss_ = 2.09; *F*_(1, 60)_ = 0.183, *p* = 0.67]. These results were consistent with Study 1, suggesting that our findings were robust.

In RTB, the number of idioms over-stated between the winning and the losing condition was not significantly different, so the authors lump together the data to test the relationship between collective efficacy and unethical behavior. A regression analysis predicting the numbers of idioms overstated as a function of collective efficacy, controlling for competition outcome, positive affect, negative affect, and team identification, revealed a significantly negative effect of collective efficacy, *b* = −0.81, *p* < 0.001, supporting Hypothesis 3. This finding contributes to literature by emphasizing the role of collective efficacy in inhibiting unethical behavior in team-based competitions. Moreover, results also showed a significantly positive effect of team identification, *b* = 0.50, *p* < 0.05, suggesting that the more individuals are identified with their team the more likely they are willing to help their team through unethical means. Thus this finding seems to be in concordance with past research on unethical pro-organizational behaviors (Umphress et al., 2010).

## Study 3

### Methods

#### Participants

We selected work groups in bank subsidiaries because they fit the definition of teams as ongoing groups imbedded in an organizational system whose members exhibit interdependencies with respect to workflow, goals, and outcomes (Kozlowski and Ilgen, 2006). The relations between bank subsidiaries also fit the notion of competition or rivalry characterized as repeated competition for market share (Kilduff et al., 2010). The data for the study were collected by means of self-administered questionnaires delivered in person to employees who were assured that their responses would be anonymous. Of the 160 questionnaires delivered, 159 usable ones were returned (45.3% male, *M*_age_ = 34.68). All had a university degree.

#### Measures

Respondents’ unethical behaviors were assessed with six items drawn from the unethical pro-organizational behavior scale (Umphress et al., 2010), α = 0.91 (e.g., “If it would help my organization, I would misrepresent the truth to make my organization look good”). Team identification (α = 0.91) and collective efficacy (α = 0.89) were same as the ones used in Study 2. Following prior literature (Kish-Gephart et al., 2010; Umphress et al., 2010; Thau et al., 2015), we also included following control variables: self-control, job satisfaction, and impression management. Self-control scale was assessed via scale by Tangney et al. (2004), α = 0.78 (e.g., “I am able to work effectively toward long-term goals”). Job satisfaction was assessed with four items drawn from a job satisfaction scale (Brooke et al., 1988), α = 0.84 (e.g., “I feel fairly satisfied with my present job”). Impression management subscale was from the Balanced Inventory of Desirable Responding (Paulhus, 1984), α = 0.86 (e.g., “Once in a while I laugh at a dirty joke”). In addition, we measured respondents’ competitiveness, perceived organizational support, and perceived market performance, as these variables could also affect their unethical behaviors in favor of their team. Competitiveness was measured by five items from the competitive orientation scale (Chen et al., 2011), α = 0.71 (e.g., “I feel somewhat disappointed when others perform better than me”). Perceived organizational support was assessed with six items (Eisenberger et al., 2001), α = 0.88 (e.g., “Our bank really cares about my wellbeing”). All responses to these scales were given on a seven-point scale ranging from 1 (*strongly disagree*) to 7 (*strongly agree*). We also controlled for organizational performance by using three items (marketing/profitability/market share) from a perceived market performance scale (Delaney and Huselid, 1996) which concerned respondents’ perceptions of their organization’s performance relative to their competitors. Responses to this scale were given on a four-point scale ranging from 1 (*worse*) to 4 (*much better*), α = 80.

### Results

Regression analysis was used to test Hypotheses 3. Results were shown in Table 1. When controlling for job satisfaction, competitiveness, perceived organizational support, impression management, perceived market performance, as well as demographic variables (gender and age), results revealed a significantly negative effect of collective efficacy, *b* = −0.39, *p* < 0.05. This finding supports Hypothesis 3 that there is a negative association between collective efficacy and unethical behavior in group-based competition. This finding is consistent with Study 2, and provides further insight for the role of collective efficacy belief in constraining unethical behavior in team-based competition.

**TABLE 1:** Study 3: regression results.

	Unethical behavior	
Variables	*b*	*SE*
(Constant)	3.71**	1.29
Gender	0.24	0.21
Age	−0.06***	0.01
Job satisfaction	−0.04	0.16
Competitiveness	0.22	0.13
Perceived organizational support	0.38*	0.19
Impression management	−0.15	0.19
Perceived market performance	0.33	0.21
Collective efficacy	−0.39*	0.19
*F*	4.69***	
*R*	0.45	
*R* ^2^	0.20	
Adjusted *R*^2^	0.16	

N = 159. Dependent variable is unethical behavior. *p < 0.05. **p < 0.01. ***p < 0.001.

## General discussion

Past decades have witnessed a growing body of research contributing to our knowledge about the antecedents and consequences of unethical behavior (Treviño, 1986; Ford and Richardson, 1994; O’Fallon and Butterfield, 2005; Tenbrunsel and Smith-Crowe, 2008; Bazerman and Gino, 2012; Treviño et al., 2014). However, empirical research examining unethical behavior in group-based competitions is limited. The present research provides insights concerning how and why group-based competition experiences shape members’ unethical behavior. We showed that there are less unethical behaviors in the group-based competition than in the individual-based competition, and that collective efficacy has the effect of curbing unethical behavior in group-based competition.

Our studies make the following theoretical contributions to literature: Research suggests that goal settings can stimulate unethical behavior (Ordóñez et al., 2009), particularly when people fail in attaining their goals (Schweitzer et al., 2004). Our studies confirmed this goal-induced unethical behaviors in individual-based competition, and more importantly, we shed further light on psychological constraints (i.e., collective efficacy belief) on unethical behavior in group-based competitions. Our research also contributes to competition and rivalry literature (Kilduff et al., 2010). Our behavioral experiments consist of two consecutive competitions, each with a winning and a losing outcome. This repeated competitive relationship is consistent to the defining feature of rivalry (Kilduff et al., 2010). Existing literature suggests that rivalries boost unethical behaviors (Kilduff et al., 2010). Our findings suggest that loss in a group-based competition did not result in more unethical behaviors as compared to the winning condition. Thus we provide new knowledge about how group-based rivalry influences individuals’ unethical behaviors.

In two behavioral experiments, we observed that losers in the individual-based competition were more intended to behave unethically as compared to winners. Few study has aimed at revealing how competition loss (vs. win) might affect unethical behavior. Our study is consistent with a related stream of research showing that individual-level financial deprivation (Kern and Chugh, 2009; Reinders Folmer and De Cremer, 2012; Sharma et al., 2014) and perceived payment inequity (Greenberg, 1990, 2002; Gino and Pierce, 2009, 2010a,2010b; Houser et al., 2012; John et al., 2014) lead to maleficent acts. Both competition loss and relative deprivation are forms of goal failure that may lead to heightened motive to restore advantages among those who are falling behind (Locke and Latham, 1990). These individuals tend to place greater significance on competition outcomes, leading to eager styles of goal pursuit (Kilduff, 2014; Converse and Reinhard, 2016; Kilduff and Galinsky, 2017) and unethical acts aiming at outperforming their opponents (Kilduff et al., 2016; Kim and Guinote, 2021). Thus our finding is consistent with goal-induced unethical behavior observed in prior studies (Schweitzer et al., 2004; Ordóñez et al., 2009; Welsh and Ordóñez, 2014).

In Study 1 and 2, different from the individual-based competition, we found that loss (vs. win) in the group-based competition did not lead to more unethical behaviors in a subsequent group-based competition. However, it is unclear whether experiencing a group-based competition would affect unethical behavior in favor of personal benefits in a subsequent individual-based competition. Therefore, we conducted a follow-up experiment in which all participants engaged in a team-based competition, followed by an individual-based competition in which each participant competed with an individual from the competing team. Experimental protocol was identical to the one used in Study 2 with the following exception: The first competition (cognitive task) was a team-based competition and the second one was an individual-based competition in which each participant competed with an individual from the competing team. We speculated that losers in the first competitive task (the team-based competition) are more unethical than winners in the consecutive individual-based competition. A total of 64 students (47% male; *M*_age_ = 19.69, *SD*_age_ = 1.42) were randomly assigned to a losing (*N* = 32) or a winning (*N* = 32) condition. The experiment was a between-subjects design. The number of idioms participants over-reported in the losing condition (*M*_loss_ = 2.13, *SD* = 2.550) was larger than those in the winning condition [*M*_win_ = 0.84, *SD* = 1.273, *F*_(1, 62)_ = 6.469, *p* = 0.013, η_p_^2^ = 0.094]. This result confirmed our speculation and suggest that group-based competition experience did not alter unethical behavior for the personal interests in the individual-based competition.

Competitions and rivalries are ubiquitous in organizational life, but it is far from clear how group-based competition processes influence employees’ unethical behaviors. Some literature points to the relation between perceived loafing and lesser effort in groups (Schnake, 1991; Mulvey and Klein, 1998; Heuzé and Brunel, 2003), while other literature suggests that engaging in group-based competitions or rivalries may promote members’ intrinsic motivation in group tasks (Tauer and Harackiewicz, 2004), motivating their cooperation with partners (Erev et al., 1993; Bornstein et al., 2002; De Cremer and van Dijk, 2002), and improving group performance (Mulvey and Ribbens, 1999; Kilduff et al., 2010; Pike et al., 2018). Our research suggests that collective efficacy belief developed in group-based competition would increase members’ confidence in achieving good performance, and such collective efficacy belief would have the effect of inhibiting unethical behaviors. Thus managers could use techniques (e.g., team-based incentives) to promote employees’ collective efficacy.

The present study has some limitations: Our research did not answer how intensity of competition affects unethical behaviors. Cartwright and Menezes (2014) interestingly showed in their experiment that competition intensity at medium level is more likely to produce unethical behavior. It is far from clear the mechanism underlying their finding, thus future research could devote to exploring how and why different levels of competitions intensity would influence unethical behaviors, especially in group-based competitions. Another limitation relates to the fact that our research did not consider whether degree of similarity between current competitive interactions and past ones would affect unethical behavior. Rivalry relational schemata develop from past competitive experience, and high degree of similarity between current competitive interactions and past ones will facilitate the activation of rivalry relational schema, and will evoke unethical behaviors to gain advantages (Kilduff, 2014; Converse and Reinhard, 2016). Future research can manipulate degree of similarity to see how it influences unethical behaviors.

## Data availability statement

The raw data supporting the conclusions of this article will be made available by the authors, without undue reservation.

## Ethics statement

The studies involving humans were approved by the Nanjing Xiaozhuang University Institutional Review Board. The studies were conducted in accordance with the local legislation and institutional requirements. The participants provided their written informed consent to participate in this study.

## Author contributions

YZ: Conceptualization, Data curation, Formal analysis, Writing – original draft. LZ: Methodology, Data curation, Writing – review and editing. YH: Data curation, Formal analysis, Writing – review and editing.
